# Anemia is a novel predictor for clinical ISR following PCI

**DOI:** 10.1186/s43044-021-00163-8

**Published:** 2021-05-01

**Authors:** Ahmed Hussein, Mohammad Shafiq Awad, Ahlam M. Sabra, Hossam Eldin M. Mahmoud

**Affiliations:** 1 Department of Internal Medicine, Faculty of Medicine, Sohag University, Nasser City, Sohag 82524 Egypt; 2 Department of Cardiology, Faculty of Medicine, Beni Suef University, Beni Suef City, 62511 Egypt; 3 Department of Internal Medicine, Faculty of Medicine, South Valley University, Qena City, Qena 83511 Egypt

**Keywords:** Anemia, PCI, MACE, Clinical ISR

## Abstract

**Background:**

Conflicting data were found regarding the anemia’s effect on percutaneous coronary intervention (PCI) outcomes. We directed our study to investigate anemia’s effect on clinical in-stent restenosis (ISR) following PCI.

**Results:**

A prospective multi-center cohort study was performed on 470 consecutive participants undergoing elective PCI. We classified the participants into two groups: group 1 who were anemic and group 2 who were non-anemic as a control group. At 1, 3, 6, and 12 months by clinic visits, we followed up with the patients to assess anemia’s clinical ISR effect. We found that 20% of the patients undergoing PCI had anemia. Anemic patients showed a statistically significant higher rate of impaired renal function and diabetes and a higher percentage of the female gender. Multivariate regression analysis for major adverse cardiovascular events (MACEs) after adjusting for confounding factors revealed that anemia represents a more risk on MACE (adjusted hazard ratio (HR) was 4.13; 95% CI 2.35–7.94; *p* value < 0.001) and carries a higher risk upon clinical ISR (adjusted HR was 3.51; 95% CI 1.88–7.16; *p* value < 0.001) over 12 months of follow-up.

**Conclusion:**

Anemic patients going through PCI are generally females, diabetics, and have renal impairment. Anemia might be considered another indicator for clinical ISR and is fundamentally associated with an increased MACE incidence.

## Background

Patients going through PCI showed a prevalence of anemia varied from 10 to >30% [1–3]. Anemia was discovered to be related to an actual higher rate of adverse cardiovascular outcomes [4, 5]. Conflicting data were found regarding the anemia’s effect on PCI outcomes. Some studies postulated that anemia is related to more danger of MACE, myocardial infarction (MI), major bleeding, and death in patients going through PCI [1, 3, 6, 7]. Anemic patients going through PCI are often older and have associated co-morbidities and severe coronary artery disease (CAD); all of these factors are considered high risks of poor PCI’s outcomes [8, 9]. A few studies showed that post PCI’s poor outcomes were only developed in patients with a severe degree of anemia, not in those with a mild or moderate degree [2, 10]. However, other studies had recommended that anemia was not, at this point, related to increased death after adjustment for confounders [2, 11, 12]. What is more, a few studies proposed that post-PCI anemia was independently related to MACE [13, 14]. ISR is still considered one of the main complications post-PCI, representing roughly 5–10%, despite the marked improvement of the new generation drug-eluting stent (DES) [15, 16]. Data is missing concerning the relationship between anemia and ISR. Theoretically, anemia may prompt a diminished supply of oxygen, and hypoxia has been related to vascular cell proliferation and angiogenesis, which are essential for the development, maintenance, and extension of the neointimal lesions associated with ISR [17–19].

In this way, anemia may help the development of ISR post-PCI. To our knowledge, just a little research was conducted on this specific topic.

## Methods

### Aim of our study

We directed the study to investigate the impact of anemia on ISR following PCI.

### Study design

It was a prospective multi-center cohort study on 470 consecutive participants undergoing elective PCI using second-generation DES. We calculated the required sample size using *χ*^2^ tests—goodness-of-fit tests: contingency tables at effect size *w* = 0.25, *α* err prob = 0.05, power (1-*β* err prob) = 0.95, and degree of freedom = 1. The output showed the non-centrality parameter *λ* = 13, critical *χ*^2^ = 3.8414588, and the total sample size = 208 patients [20]. We added 10% drop out or loss of follow-up to have a minimum total sample size = 229.

We classified the participants into two groups: group 1 includes 94 participants, who were anemic, and group 2 includes 376 participants, who were non-anemic as a control group.

All the chose patients gave informed, written consent. We conformed the study protocol to the 1975 Declaration of Helsinki’s ethical guidelines, and the ethics committee approved it at the Sohag, Beni Suef, and Qena Faculty of Medicine.

#### Patients were subjected to

1. A detailed history and physical examination for detecting cardiovascular risk factors and the clinical presentation at the Cath. Lab. Unit.
2. Baseline complete blood count and serum hemoglobin level. We defined anemia, as a hemoglobin level for men of <13 g/dL and women <12 g/dL [21].
3. Resting 12 lead electrocardiogram to detect abnormal ST-segment or T wave or cardiac arrhythmia.
4. An echocardiography study assessed the resting abnormal regional wall motion and the left ventricle ejection fraction (LVEF) by Simpson’s rule.
5. PCI strategy and the devices utilized were at the operator’s choice. The participants received a dual antiplatelet treatment comprising a peri-procedure aspirin dose of 300 mg and post-procedure dose of 100 mg/day and a clopidogrel loading dose of 300–600 mg and a post-procedure dose of 75 mg/day or ticagrelor 90 mg given twice daily for at least 1 year.

#### Follow-up

At 1, 3, 6, and 12 months by clinic visits, we followed up with the patients. The assessed endpoints were MACEs that incorporate (CABG, TLR, TVR, CVS, MI, major bleeding, and cardiac and non-cardiac death) and clinical ISR over 12 months. We defined MI by the third universal definition of MI [22]. Clinical ISR was defined as a necessity for reintervention due to ischemia burden and required both an evaluation of luminal stenosis beside the patient’s clinical setting. We quantified bleeding by the definition criteria of the Bleeding Academic Research Consortium [23]. TVR and TLR included CABG. MACE was calculated by the number of patients who developed adverse outcomes in each group.

### Participant characteristics

All patients ≥ 18 years with de novo CAD candidates for elective PCI using DES, who accepted to partake in the study were included. We excluded those with a chronic total occlusion lesion, left main CAD, who need primary PCI; end-stage renal disease; decompensated liver disease; or malignancy.

### Analysis of data

We analyzed data using SPSS version 20. We used the Student *t* test to compare the two groups’ means, while the chi-square test was used to compare the qualitative data. When the expected cell count is < 5, Fisher’s exact correction was used. We analyzed the impact of anemia on PCI outcomes using Cox regression analyses after adjusting for confounding factors for MACEs and clinical ISR over 12 months. Covariables adjusted in the multivariate Cox regression analyses included baseline demographics and clinical characteristics (age, sex, BMI, dyslipidemia, DM, hypertension, smoking, and family history of CAD), baseline clinical presentations (stable angina, unstable angina, and NSTEMI), baseline laboratory parameters (creatinine clearance rate (Ccr) and LVEF), and baseline lesion and procedural characteristics (no. of stenosed vessels, incidence of bifurcation intervention, no. of inserted stents, stent diameter, stent length, inflation pressure, the use of post-stent balloon dilatation, and TIMI flow). *p* value was significant ≤ 0.05.

## Results

### Baseline patient characteristics

We found that 20% (94 out of 470) of the patients undergoing PCI were anemic. The mean hemoglobin level in anemic patients was 9.37±1.51 versus 14.17±0.59 gm/dL in the non-anemic group. Patients with anemia showed a statistically significant higher percentage of female gender and diabetes and a statistically significant lower percentage of smokers. Also, the incidence of impaired renal function was significantly higher in anemic than non-anemic participants, *p* value <0.001 (Tables 1, 2, and 3)

**Table 1:** Baseline demographics and clinical characteristics

Variables	Group 1 (anemic patients), *N*=94	Group 2 (non-anemic), *N*=376	*p* value
**Age**			
Mean ± SD (years)	50.43±10.54	51.46± 10.87	0.557
**Sex**			
Male	46 (48.94%)	262 (69.68%)	< 0.001
Female	48 (51.06%)	114 (30.32%)	
**Body mass index, kg/m**^**2**^			
Mean± SD	25.91±3.60	26.44±3.11	0.363
**Dyslipidemia**			
Yes	48 (51.06%)	196 (52.13%)	0.782
**Diabetes mellitus**			
Yes	66 (70.21%)	198 (52.66%)	0.002
**Hypertension**			
Yes	50 (53.19%)	216 (57.45%)	0.457
**Smoking**			
Yes	38 (40.43%)	202 (53.72%)	0.021
**Family history of CAD**			
Positive	22 (23.40%)	96 (25.53%)	0.670

*Abbreviations*: *CAD* coronary artery disease, *kg/m*^*2*^ kilogram/meter square, *N* number, *SD* standard deviation, *%* percentage

**Table 2:** Baseline clinical presentations

Variables	Group 1	Group 2	*p* value
**Unstable angina**			
Yes	38 (40.43%)	162 (43.09%)	0.641
**NSTEMI**			
Yes	42 (44.68%)	152 (40.42%)	0.454
**Stable angina**			
Yes	14 (14.89%)	62 (16.49%)	0.707

*Abbreviations*: *NSTEMI* non-ST elevation myocardial infarction

**Table 3:** Baseline laboratory parameters

Variables	Group 1	Group 2	*p* value
**Hemoglobin level**			
Mean± SD (g/dL)	9.37±1.51	14.17±0.59	<0.001
**Serum LDL**			
Mean± SD (mg/dL)	136.13±30.56	133.88±30.13	0.649
**Ccr**			
<60 mL/min/1.73m^2^(%)	8 (8.51%)	4 (1.06%)	<0.001
**Hemoglobin A1c**			
Mean± SD (%)	7.17±1.86	6.59±1.63	0.037
**LVEF**			
Mean± SD (%)	55.11 ± 5.79	54.87 ± 5.80	0.800

*Abbreviations*: *Ccr* creatinine clearance rate, *g/dL* gram/deciliter, *Hemoglobin A1c* glycated hemoglobin, *LDL* low-density lipoprotein, *mg/dL* milligram/ deciliter, *LVEF* left ventricle ejection fraction

### Baseline lesion and procedural characteristics

Anemic patients showed a significantly higher mean of the implanted stent’s length (27.11 ± 6.13 vs. 24.89 ± 6.07 mm, *p* value = 0.027) and higher incidence of use of post-stent balloon dilatation (15.96% vs. 8.52%, *p* value = 0.031) than non-anemic (Table 4).

**Table 4:** Baseline lesion and procedural characteristics

Variables	Group 1	Group 2	*p* value
**No. of stenosed vessels**			
One vessel	50 (53.19%)	196 (52.13%)	0.880
Two vessels	28 (29.79%)	120 (31.91%)	
Multiple vessels	16 (17.02%)	60 (15.96%)	
**Incidence of bifurcation intervention**	11 (10.34%)	24 (6.38%)	0.079
**No. of inserted stents**			
Single stent	38 (40.43%)	162 (43.09%)	0.651
Two stents	34 (36.17%)	142 (37.76%)	
Three or more stents	22 (23.40%)	72 (19.15%)	
**Stent diameter**			
Mean ± SD (mm)	3.02 ± 0.35	3.11 ± 0.33	0.078
**Stent length**			
Mean± SD (mm)	27.11 ± 6.13	24.89 ± 6.07	0.027
**Inflation pressure**			
Mean± SD (atm)	14.06 ± 2.68	14.87 ± 2.64	0.064
**Use of post-stent balloon dilatation**	15 (15.96%)	32 (8.52%)	0.031
**TIMI flow**			
Grade 2	14 (14.89%)	42 (11.17%)	0.319
Grade 3	80 (85.11%)	334 (88.83%)	

*Abbreviations*: *atm* atmosphere, *mm* millimeter, *No.* number, *SD* standard deviation, *TIMI* thrombolysis in myocardial infarction

### Clinical outcomes at 12 months follow-up

A significantly higher MACE incidence was observed in anemic patients than in non-anemic (27.66% versus 7.45%, *p* value <0.001). TLR, TVR, MI, and cardiac death showed a significantly higher incidence in anemic patients. After adjusting for confounders, the multivariate regression analyses for MACEs revealed that anemia represents a higher risk upon MACEs over 12 months (adjusted HR was 4.13; 95% CI 2.35–7.94; *p* value < 0.001) (Tables 5 and 6).

**Table 5:** Cumulative incidence of MACEs over 12 months of follow-up period

Variables	Group 1	Group 2	*p* value
**Major bleeding**			
Yes	0	0	
**CVS**			
Yes	0	0	
**MI**			
Yes	6 (6.38%)	2 (0.53%)	0.001
**TVR**			
Yes	22 (23.40%)	22 (5.85%)	<0.001
**TLR**			
Yes	16 (17.02%)	20 (5.32%)	0.001
**CABG**			
Yes	2 (2.13%)	4 (1.06%)	0.345
**Cardiac death**			
Yes	4 (4.26%)	2 (0.53%)	0.016
**Non-cardiac death**			
Yes	0	0	
**MACEs**			
Yes	26 (27.66%)	28 (7.45%)	<0.001

*Abbreviations*: *CABG* coronary artery bypass graft, *CVS* cerebrovascular stroke, *MACEs* major adverse cardiovascular events, *MI* myocardial infarction, *TLR* target lesion revascularization, *TVR* target vessel revascularization

**Table 6:** Multivariate Cox hazard regression analyses for MACEs over 12 months of follow-up

Variable	Unadjusted HR ratio (95% CI) for anemic patients versus non-anemic (reference)			Adjusted HR ratio (95% CI) for anemic patients versus non-anemic (reference)		
	HR	95% CI	*p* value	HR	95% CI	*p* value
**MACEs**	3.011	1.13–7.22	0.009	4.13	2.35–7.94	< 0.001

*Abbreviations*: *CI* confidence interval, *HR* hazard ratio, *MACEs* major adverse cardiovascular events

The clinical ISR showed a statistically significant higher cumulative incidence over 12 months in anemic patients (17.02% versus 4.79%, *p* value <0.001). After adjusting the confounding factors, the multivariate Cox regression analyses for clinical ISR showed that anemia carries a higher risk upon clinical ISR over 12 months (adjusted HR was 3.51; 95% CI 1.88–7.16; *p* value < 0.001). (Tables 7 and 8)

**Table 7:** Cumulative incidence of clinical ISR over 12 months of follow-up period

Variables	Group 1	Group 2	*p* value
**Clinical ISR** (documented by coronary angiography)			
Yes	16 (17.02%)	18 (4.79%)	<0.001

*Abbreviations*: *ISR* in-stent restenosis

**Table 8:** Multivariate Cox hazard regression analyses for clinical ISR over 12 months of follow-up

Variable	Unadjusted HR ratio (95% CI) for anemic patients versus non-anemic (reference)			Adjusted HR ratio (95% CI) for anemic patients versus non-anemic (reference)		
	HR	95% CI	*p* value	HR	95% CI	*p* value
**Clinical ISR** (documented by coronary angiography)	6.23	1.97–22.92	0.005	3.51	1.88–7.16	< 0.001

*Abbreviations*: *CI* confidence interval, *HR* hazard ratio, *ISR* in-stent restenosis

## Discussion

Our study found that 20% of the patients undergoing PCI were anemic with a mean hemoglobin level of 9.37±1.51 gm/dL. This finding matched the results of Jiang L et al.’s study [24], which found that anemia’s prevalence was 12.5% pre-PCI and 29.0% post-PCI. Many other studies showed a prevalence of anemia varying from 10 to 30% in patients going through PCI [1–3, 7, 13].

We observed that the percentage of the female gender, diabetes, and renal impairment was significantly higher in anemic patients. In contrast, the percentage of smokers was significantly lower in anemic patients. These findings matched with Jiang et al.’s study [24], who conducted a large study on 10,658 Chinese patients undergoing PCI and reported that anemic patients undergoing PCI had a significantly higher incidence of diabetes, renal impairment, old age, acute coronary syndrome, and severe CAD. A comparable result came from Hosseini et al. [10], who observed a higher incidence of the female gender, old age, and renal failure in anemic patients undergoing PCI. Much other research has shown similar results [2, 3, 7, 13, 14].

We found a significantly higher incidence of MI, TLR, TVR, cardiac death, and MACEs in anemic patients following PCI. We performed a multivariate regression analysis for MACEs after adjusting the confounders and observed that anemia carries a higher risk upon MACEs at 12 months. Conflicting results were found regarding the impact of anemia on PCI outcomes. A meta-analysis conducted by Kwok et al. [1] investigated the adverse outcomes and mortality in anemic patients following PCI and collected data from 44 studies with 230,795 participants. This meta-analysis showed that the prevalence of anemia in patients going through PCI was 16%, with a significantly increased risk of MI, mortality, bleeding, and MACE. Also, Jiang et al. [24] showed an increased incidence of bleeding and cerebrovascular stroke in patients with pre-PCI anemia and a higher incidence of bleeding, MI, TVR, MACE, and all-cause death in post-PCI anemic patients over 2-year follow-up. While Rathod et al. [2] studied the impact of anemia on the outcomes following primary PCI, they found that 19% of the patients were anemic with a higher incidence of diabetes, hypertension, dyslipidemia, cardiogenic shock, previous MI, and previous PCI. They found a significantly higher all-cause mortality in anemic patients in the presence of other risk factors after a 3-year follow-up period. However, after adjusting for the other risk variables, the multivariate regression analysis showed that anemia had no significant impact on mortality or MACE. Other studies showed similar results to Rathod et al. [11, 12].

After adjusting the confounding factors in our study, the multivariate regression analyses for clinical ISR over 12 months showed that anemia was an independent predictor for clinical ISR. The diminished supply of oxygen might explain this finding in the presence of anemia. Hypoxia has been related to vascular cell proliferation and angiogenesis, essential for developing, maintaining, and extending the neointimal lesions associated with ISR [17–19]. Another support to our result came from Giustino et al. [25], who demonstrated that anemia is independently correlated with increased platelet reactivity in patients going through DES PCI, which have been shown to add to the development of ISR [26, 27]. Geng et al. [28] found that the baseline red blood cell distribution width (a common finding in the anemic patient) was significantly related to ISR at follow-up.

Our research has some limitations to be considered. First, we did not think about some potential confounders that may affect the PCI outcomes as the severity and complexity of CAD, the use of IVUS to optimize stent expansion and apposition, the type of stent platform in each group, some co-morbidities as heart failure and peripheral arterial disease, the patient’s lifestyle, prescribed drug regimens, and medication adherence. Second, we only considered periprocedural serum hemoglobin. Finally, we do not know about the long-term outcome beyond 1 year.

## Conclusion

Anemic patients undergoing PCI are mostly females, diabetics, and have renal impairment. Anemia may be considered a novel predictor for clinical ISR and significantly associated with increased MACE incidence.

## Data Availability

The datasets used and analyzed during the current study are available from the corresponding author on reasonable request.

## Abbreviations

CABG: Coronary artery bypass graft
CAD: Coronary artery disease
CVS: Cerebrovascular stroke
DES: Drug-eluting stent
ISR: In-stent restenosis
LDL: Low-density lipoprotein
MACE: Major adverse cardiovascular event
MI: Myocardial infarction
PCI: Percutaneous coronary intervention
TLR: Target lesion revascularization
TVR: Target vessel revascularization
